# Functional analysis of apple stem pitting virus coat protein variants

**DOI:** 10.1186/s12985-019-1126-8

**Published:** 2019-02-08

**Authors:** Xiaofang Ma, Ni Hong, Peter Moffett, Yijun Zhou, Guoping Wang

**Affiliations:** 1 State Key Laboratory of Agricultural Microbiology, Huazhong Agricultural University, Wuhan, Hubei 430070 People’s Republic of China; 2 Key Laboratory of Plant Pathology of Hubei Province, College of Plant Science and Technology, Huazhong Agricultural University, Wuhan, Hubei 430070 People’s Republic of China; 3 Centre SÈVE, Département de Biologie, Université de Sherbrooke, 2500 Blvd. de l’Université, Sherbrooke, QC J1K 2R1 Canada; 4 Jiangsu Academy of Agricultural Sciences, Key Lab of Food Quality and Safety of Jiangsu Province-State Key Laboratory Breeding Base, Institute of Plant Protection, Nanjing, 210014 China

**Keywords:** Apple stem pitting virus, Coat protein, CP variants, Aggregate, RNA silencing suppressor

## Abstract

**Background:**

Although the canonical function of viral coat protein (CP) is to encapsidate the viral genome, they have come to be recognized as multifunctional proteins, involved in almost every stage of the viral infection cycle. However, CP functions of Apple stem pitting virus (ASPV) has not been comprehensively documented. This study aimed to characterize the functions of ASPV CP and any functional diversification caused by sequence diversity of six ASPV CP variants and studied their biological, serological, pathogenic and viral suppressor of RNA silencing (VSR) functions.

**Methods:**

Six ASPV CP variants that have previously been shown to belong to different subgroups were selected here to study their diversity functions. Agrobacterium mediated infiltration (Agroinfiltration) was used to express YFP-ASPV-CPs in *Nicotiana. benthamiana* and infect *Nicotiana. occidental* with PVX-ASPV-CPs in*.* Confocal microscopy was used to detect YFP-ASPV-CPs florescence. CPs expressed in *Escherichia coli* BL21 (DE3) were induced by IPTG.

**Results:**

In this study, we showed that recombinant CPs expressed in *Escherichia coli* BL21 (DE3) had different levels of serological reactivity to three anti-ASPV antibodies used to detect ASPV. Furthermore, fusion CPs with YFP (YFP-CPs) expressed in *N. benthamiana* cells differed in their ability to form aggregates. We also showed that ASPV isolates that harbour these CPs induced different biological symptoms on its herbaceous host *N. occidentalis*. At the same time, we found that all six CPs when expressed in PVX vector showed similar VSR activity and produced similar symptoms in *N. occidentalis*, despite their differences in amino acids.

**Conclusions:**

Different ASPV isolates induced different symptoms in *N. occidentalis*, however, ASPV CP variants expressed in PVX vector showed the same symptoms in *N. occidentalis* plants. Also, we showed that ASPV CP variants has the same level of VSR activity, but they have different abilities to aggregate in *N. benthamiana*.

**Electronic supplementary material:**

The online version of this article (10.1186/s12985-019-1126-8) contains supplementary material, which is available to authorized users.

## Background

Apple stem pitting virus (ASPV) is the type species of the *Foveavirus* genus in the *Betaflexiviridae* family [1]. It possesses a single stranded positive RNA (+ssRNA) genome comprising of approximately 9300 nucleotides (nts), which encodes five open reading frames (ORFs, ORF1-ORF5) as well as the 5′ untranslated region (UTR) and 3’ UTR. ORF1 encodes the viral RNA-dependent RNA polymerase (RdRP), ORF2-ORF4 encode triple gene block proteins (TGBp1-TGBp3) and ORF5 encodes the viral coat (capsid) protein (CP) [2]. ASPV infects several plant species and causes a wide range of symptoms from symptomless to xylem pits, epinasty, decline, vein yellowing, leaf red mottling, pear necrotic spot or fruit stony pits depending on the plant species, the cultivar and the viral strain/isolate [2–5].

It has been shown that when a virus adapts to a new host, variation is primarily manifested as amino acids substitutions, which allows virus entry into the new host efficiently, blocks interactions with host proteins or allows the virus to circumvent immunity in both the new and the old host [6–8]. The RdRP encoded by many RNA viruses are known to be error-prone, and this error-prone replication is thought to be important for viruses to generate a pool of different progeny genomes to adapt to potentially diverse new hosts [9]. Several studies have shown that each ASPV ORF possesses a high degree of genetic variability between/within isolates [4, 5, 10–13], especially in the CP-encoding ORF. Our previous study showed that ASPV CP variants could be divided into three groups (pear group, apple group and Korla pear), which correlated with their isolated hosts [13]. Furthermore, variants from pear isolates could be divided into six subgroups (subgroup A-F), and CP variants from a different subgroup have a different CP size because of amino acid insertions or deletions in the N terminal portion of CP [13]. These previous observations might imply host-driven adaptations have affected genetic diversification of ASPV CP variants.

In addition to mediating encapsidation and protecting the viral genome from degradation, multiple reports have shown that viral CPs play multiple functions, including roles in viral RNA translation, viral RNA replication, viral movement, activation of host immune, RNA binding, virus transmission, symptom development, and viral suppressor of RNA silencing (VSR) [14]. TGBp1 proteins, encoded by several viruses in the genus *Potexvirus*, have been shown to have VSR activity [15, 16]. *Potexvirus* and *Foveavirus* are phylogenetically related viruses, both of which encoded potex-like TGB proteins as viral movement protein [17]. TGBp1 encoded by TGB-encoding viruses were clustered into two major groups by phylogenetic analysis of the NTPase/helicase (which is a conserved domain) sequences of TGBp1, filamentous viruses (genera *Potexvirus*, *Carlavirus*, *Foveavirus* and *Allexivirus*) and rod-shaped viruses (genera *Hordeivirus*, *Benyvirus*, *Pomovirus* and *Pecluvirus*) [18]. However, none of those functions have been explored with respect to ASPV: whether TGBp1 encoded by ASPV functional as a VSR or whether the diversity of symptoms induced by ASPV in its natural hosts is due to the genetic diversity of its genome, including the CP. In this study, we selected six CP sequences belonging to different subgroups including HB-HN1–3/subgroup A, HB-HN7–18/subgroup B, YN-MRS-17/subgroup D, HB-HN6–8/subgroup E, HB-HN9–3/subgroup F, LN-AP-1/apple group [13], to test whether differences in CP amino acid sequences could result in changes in symptomatology, VSR activity, and serological reactivity.

## Methods

### Plant growth conditions and virus inoculation

*Nicotiana. benthamiana* and *Nicotiana. occidentalis* plants were grown on soil (BM6, Berger) in growth chambers with 16-h-light/8-h-dark photoperiod at 25 °C. ASPV isolates were confirmed by Reverse Transcription -Polymerase Chain Reaction (RT-PCR) in previous study [13]. Infections of 3-week-old *N. occidentalis* plants were performed by rub inoculation as previously described in our lab [19]. Saps were produced from ASPV infected apple or pear plants by grinding infected leaf tissue in the buffer containing 0.02 MPB (1 mM Na_2_HPO_4_.12H_2_O, 0.5 mM NaH_2_PO_4_.2HO_2_), 0.15% β-Mercaptoethanol, 0.45% DIECA, pH 7.4.

### Total RNA extraction and RT-PCR

Total RNA was extracted from 0.1 g of leaf tissue using cetyltriethylammonium bromide (CTAB) [20] and was subsequently used as a template for ASPV detection by RT-PCR [13]. The primers used for ASPV detection were (Menzel et al. 2002): 370-F: 5’-ATGTCTGGAACCTCATGCTGCAA-3′/370-R: 5’-TTGGGATCAACTTTACTAAAAAGCATAA-3′. First-strand cDNA synthesis was performed using 0.5 mM of random hexamers (TaKaRa, Dalian, China) and M-MLV reverse transcriptase (Promega, Madison, USA) at 37 °C for 1.5 h. PCRs were performed in a 25 μL volume with reaction mixtures containing 2.5 μL 10 × PCR buffer, 0.5 mM dNTP, 1 mM specific primer, 0.15 μL of 5 U/lL rTaq DNA polymerase (TaKaRa, Dalian, China), and 3 μL first-strand cDNA as templates.

### Vector construction

Based on phylogenetic analysis of the ASPV CP gene in our previous study [13], unique CP sequences (clones) from five pear and one apple ASPV isolates (HB-HN1–3, HB-HN7–18, HB-HN6–8, HB-HN9–3, YN-MRS-17 and LN-AP-1) were selected to produce recombinant proteins to be used for analysis of electrophoretic mobility, serological reactivity and VSR activity. For generation of different versions of CP constructs, pMD18-T-CP constructs were used as templates for PCR amplification using rTaq DNA polymerase (TaKaRa, Dalian, China). All primer sequences used in this study are listed in Additional file 1: Table S1 PCR fragments were cloned into the pMD18-T simple vector (TaKaRa, Dalian, China) for sequencing.

For expression of ASPV-CPs fused with a His tag in *Escherichia coli* (*E. coli*) BL21 (DE3), HB-HN9–3 was cloned into pET-28a (+) (Novagen, Madison, WI, USA) by double digestion with *Bam*HI and *Hin*dIII, HB-HN1–3, HB-HN7–18, HB-HN6–8, LN-AP-1 and YN-MRS-17 were cloned into pET-28a (+) by double digestion with *Sac*I and *Sal*I. The recombinant expression vectors were named as pET-HB-HN1–3, pET-HB-HN7–18, pET-HB-HN6–8, pET-HB-HN9–3, pET-YN-MRS-17 and pET-LN1-AP-1, respectively.

To generate PVX-ASPV-CP vectors expressing CPs in *N. benthamiana* and *N. occidentalis*, CPs were cloned into *Cla*I and *Sal*I sites of the Potato Virus X (PVX) vector pGR106; these vectors were named PVX-HB-HN1–3/HB-HN7–18/HB-HN6–8/HB-HN9–3/YN-MRS-17/LN-AP-1, respectively. The PVX expression construct pGR106 (Peart et al. 2002), 35S:P25, 35S:mGFP5 and 35:P19 [15] have been previously described.

For generation of pEAQ-YFP-CP vectors expressing YFP-CPs in *N. benthamiana,* CPs were cloned into *Xba*I and *Sal*I sites of a modified vector pEAQ-SE [21].

### Recombinant ASPV CP (rCP) expression in *E. coli BL21* (DE3)

pET-28a-ASPV-CP constructs were transformed into *E. coli* BL21 (DE3). Recombinant CP (rCP) expression was induced in Luria-Bertani (LB) medium containing 50 mg/L kanamycin and 1 mM/L isopropyl-β-d-thiogalactoside (IPTG) at 30 °C for 6 h, and then evaluated by 10.5% SDS-PAGE. SDS-PAGE Gels were stained with 0.25% Coomassie blue G250 solution.

### Preparation of antiserum against ASPV rCP and Western blot

Antiserum against ASPV rCP expressed by pET-HB-HN6–8, pET-HB-HN9–3 and pET-YN-MRS-17 were prepared and purified based on methods reported previously [19, 22] and named PAb-HB-HN6–8, PAb-HB-HN9–3, PAb-YN-MRS-17, respectively. Western blotting was used to detect CPs expressed in *E. coli* with the three antibodies. For western blot, total induced proteins from *E. coli* cells were separated on 10.5% resolving gels and transferred onto a polyvinylidene difluoride (PVDF) membrane (BioRad, USA), followed by blocking with 5% (*w*/*v*) skimmed milk powder in 1x PBST (0.01 M PBS, 0.05–0.1% Tween-20, pH 7.4) at 37 °C for 2 h or 4 °C overnight. Membranes were subsequently incubated with purified primary antibodies at dilutions of 1:500 and then incubated with secondary antibody alkaline phosphatase (AP) conjugated goat anti-rabbit IgG (Sigma, Germany) diluted at 1: 5000. Antigen-antibody reactions were visualized by incubation in BCIP/NBT substrate solution (Amresco, USA). Image J was used to quantify reaction signals on the blots.

### Transient expression of ASPV CPs in *N. benthamiana*

GFP-expressing transgenic *N. benthamiana* line 16 C (a generous gift from David Baulcombe, UK), *N. occidentalis* or *N. benthamiana* wildtype plants were germinated and grown in a growth chamber maintained at 26 °C with 16 h day and 8 h night. PVX-HB-HN1–3/HB-HN7–18/HB-HN6–8/HB-HN9–3/YN-MRS-17/LN-AP-1 were transformed into *Agrobacterium tumefaciens* strain GV3101. pEAQ-YFP-HB-HN1–3/HB-HN7–18/HB-HN6–8/HB-HN9–3/YN-MRS-17/LN-AP-1 were transformed to *Agrobacterium tumefaciens* strain AGL1*.* Transient expression by *Agrobacterium* mediated infiltration (agroinfiltration) was performed as previously described [23]. GFP expression was monitored under UV light using a handheld lamp (BLAK RAY, UVP).

### Immunoblotting for proteins extracting from plants

The indicated *N. benthamiana* or *N. occidentalis* leaves tissue were ground in liquid nitrogen, 1 leaf disk of the ground powder was mixed with 50 μL 1 x sample buffer (50 mM Tris-HCl pH 6.8, 6% glycerol, 2% SDS, 0.01% (*W*/*V*) Bromophenol blue, 10 mM DTT) and then incubated at 95 °C for 5 min. Protein samples from total leaf tissue extracts were separated by SDS-PAGE on 10.5% resolving gels and transferred onto PVDF membrane (BioRad, USA). For detecting PVX vector based ASPV-CPs in *N. occidentalis*, membranes were subsequently incubated with purified primary antibodies at dilutions of 1:500 and then incubated with secondary antibody alkaline phosphatase (AP) conjugated goat anti-rabbit IgG (Sigma, Germany) diluted at 1: 5000. Horseradish peroxidase (HRP) conjugated GFP antibody was used for detecting 35S:mGFP5 expressed GFP proteins (Santa Cruz; 1:3000 dilution). Anti-GFP rabbit polyclonal antibody (Genscript; 1:5000 dilution) was used to detect YFP-ASPV-CPs expressed in *N. benthamiana* followed by anti-IgG rabbit-HRP polyclonal antibodies (Genscript; 1:10,000 dilution).

### Protein size and secondary structure prediction

Protein size was predicted by online software Protein Molecular Weight (https://www.bioinformatics.org/sms/prot_mw.html). Protein secondary structure was predicted by SOPMA (https://npsa-prabi.ibcp.fr/cgi-bin/npsa_automat.pl?page=npsa_sopma.html) [24].

### Confocal imaging

*N. benthamiana* plants were agroinfiltrated with constructs to express YFP-ASPV-CPs, and samples were imaged 72 hpi on an OLYMPUS Confocal Laser Scanning Microscope FV3000 using the preset settings for YFP with excitation at 488 nm.

## Results

### Different symptoms induced in *N. occidentalis* by ASPV isolates

**Fig. 1:**
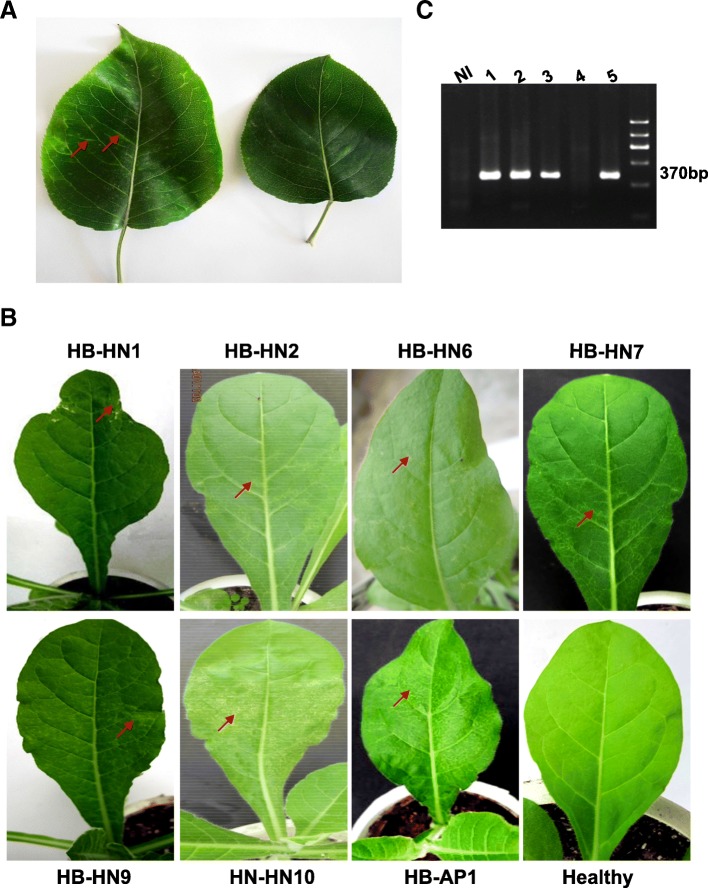
Symptoms induced by different isolates of Apple stem pitting virus (ASPV) on *Nicotiana occidentalis.* **a** Left, symptoms induced on pear leaves in an orchard in Hangzhou city, Zhejiang province, China. Right, healthy pear leaf. **b** Symptoms induced on *N. occidentalis* plants 14 days post inoculation (dpi), red arrows indicated typical symptoms in each photo. Pear isolates HB-HN1, HB-HN2, HB-HN6, HB-HN7, HB-HN9, and HB-HN10 were collected from an orchard in Wuhan city, Hubei province in China, Apple isolate HB-AP1 was collected from the same orchard. **c** Representative result of RT-PCR products to detect ASPV. NI, Non-Infected

ASPV induces yellow vein leaves (Fig. 1a left) and stone fruit on pear but does not induce symptoms on apple leaves or fruit. To better observe different symptoms induced by ASPV isolates, *N. occidentalis*, which is a well documented herbaceous host of ASPV, was used to infect with ASPV pear or apple isolates (Fig. 1c). Symptoms induced on leaves of *N. occidentalis* were photographed at 14 days post inoculation (dpi) (Fig. 1b). Different symptoms were observed, including leaf deformity (HB-HN1), Secondary veins yellowing (HB-HN2 and HB-HN7), Regular faded green circular spots (HB-HN6), Irregular faded green spots (HB-HN9), Secondary veins necrosis (HB-HN10 and LN-AP1).

### Different serological reactivity between CPs of different ASPV isolates

Six unique CP sequences (HB-HN1–3, HB-HN7–18, HB-HN6–8, HB-HN9–3, YN-MRS-17 and LN-AP1–1) belong to different groups or subgroups in the phylogenetic tree [13] (Table 1), which shared similarity ranging from 72.5 to 87.8% at nt level and 78.2 to 88.8% at the aa level (Table 2). Secondary structures prediction indicated that proteins encoded by these CP variants has different number and percentage of Alpha helix, Extended strand, Beta turn and Random coil (Table 3). Amino acids changes/secondary structure differences between ASPV CPs might result in antigen changes, which further results in difficulties in ASPV detection. To detect ASPV isolates easily, three polyclonal antibodies PAb-HB-HN9–3, PAb-HB-HN6–8 and PAb-YN-MRS-17 were produced in this study, based on three CP variants from different subgroups (HB-HN9–3/subgroup F, HB-HN6–8/subgroup E, YN-MRS-17/subgroup D).

**Table 1:** ASPV unique CP sequences/isolates used in this study

GenBank ID	Unique CP Name	Subgroup/Group	Isolate Name	Original host	Cultivar names
JX673791	HB-HN1–3	A/Gp1	HB-HN1	Pear	*P. pyrifolia* cv. Ershishiji
JX673794	HB-HN6–8	E/Gp1	HB-HN6	Pear	*P. bretschneideri* cv. Xuehuali
JX673796	HB-HN7–18	B/Gp1	HB-HN7	Pear	*P. pyrifolia* cv. Fengshui
JX673797	HB-HN9–3	F/Gp2	HB-HN9	Pear	*P. pyrifolia* cv. Fengshui
JX673789	YN-MRS-17	D/Gp1	YN-MRS	Pear	*P. pyrifolia* cv. Meirensu
JX673803	LN-AP1–1	−/Gp2	LN-AP1	Apple	Unknown
–	–	–	HB-HN10	Pear	*P. pyrifolia* cv. Fengshui
–	–	–	HB-HN2	Pear	*P. pyrifolia* cv. Ershishiji

**Table 2:** Pairwise sequence identity of nucleotide and amino acid between the six unique CP sequences

Name	HB-HN1–3	HB-HN6–8	HB-HN7–18	HN-HN9–3	LN-AP1–1	YN-MRS-17
HB-HN1–3	100%	85.5%^a^	88.8%	81.7%	83.8%	86.6%
HB-HN6–8	80.9%^b^	100%	87.1%	78.2%	81.8%	88.5%
HB-HN7–18	87.8%	80.4%	100%	82.7%	83.0%	88.7%
HN-HN9–3	74.9%	72.5%	74.9%	100%	81.5%	83.1%
LN-AP1–1	74.9%	78.9%	78.8%	74.2%	100%	83.7%
YN-MRS-17	80.9%	86.2%	81.1%	76.2%	80.6%	100%

^a^lower left and ^b^ upper right represent nucleotide and amino acid similarity between each pair of clones, respectively

**Table 3:** Secondary structure prediction of proteins encoded by the six unique CP sequences

Name	# Total Amino acids (AA)	Alpha helix #AA	Extended strand #AA	Beta turn #AA	Random coil #AA
HB-HN1–3	394	99 (25.13%)^a^	61 (15.48%)	23 (5.84%)	211 (53.55%)
HN-HN7–18	394	111 (28.17%)	50 (12.69%)	11 (2.79%)	222 (56.35%)
HB-HN6–8	410	129 (31.46%)	52 (12.68%)	20 (4.88%)	209 (50.98%)
HB-HN9–3	410	127 (30.98%)	37 (9.02%)	17 (4.15%)	229 (55.85%)
YN-MRS-17	374	134 (35.83%)	42 (11.23%)	22 (5.88%)	176 (47.06%)
LN-AP1–1	396	117 (29.55%)	60 (15.15%)	20 (5.05%)	199 (50.25%)

^a^indicated that 99 amino acids in this protein were predicted to form Alpha helix structure, which is 25.13% of the total amino acids number

**Fig. 2:**
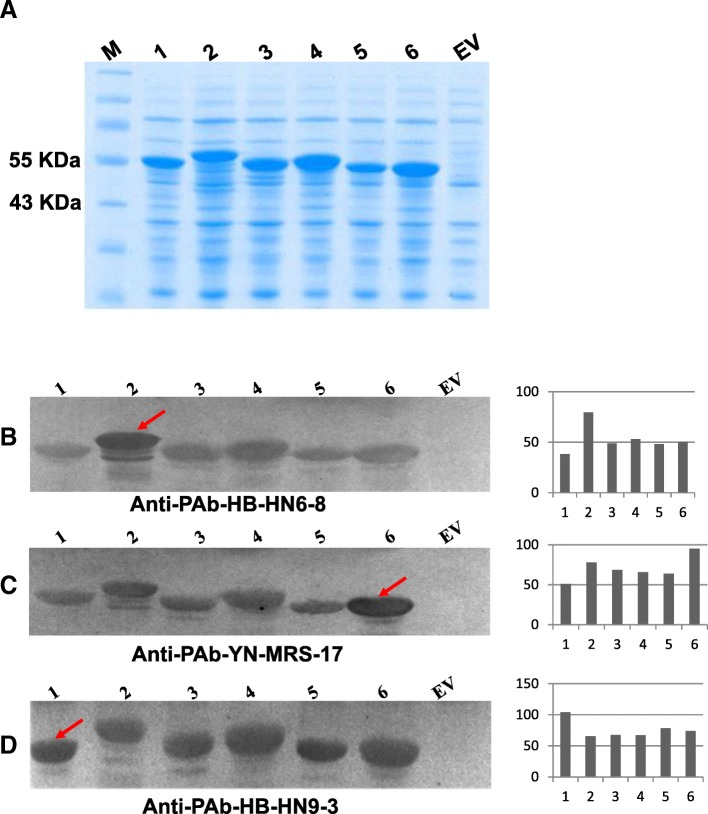
Analysis of recombinant CPs from different ASPV isolates by SDS-PAGE and western blot. **a** SDS-PAGE analyze of rCPs expressed in *E. coli*. Lane M: Protein Ladder; Lanes 1–6 and Lane EV (from left to right): protein extracts from *E. coli* transformed with vectors pET-HB-HN9–3, pET-HB-HN6–8, pET-LN-AP1–1, pET-HB-HN7–18, pET-HB-HN1–3, pET-YN-MRS-17 and the empty vector pET-28a (+), respectively. **b-d** Western blot analysis of rCPs by antibody PAb-HB-HN6–8, PAb-YN-MRS-17 and PAb-HB-HN9–3, respectively. Hybridization signals on western blots were quantified by Image J software (right column of B-D)

To test whether genetic diversity between CP variants affected serological features (antibody-antigen recognition efficiency), six recombinant CPs were expressed in *E. coli*. SDS-PAGE analysis indicated that the six rCPs were efficiently expressed and showed different migration rates (Fig. 2a). The predicted molecular sizes of these CP variants were 43.42 KDa (HB-HN9–3) > 43.31 KDa (HB-HN6–8) > 42.19 KDa (LN-AP1–1) > 42.02 KDa (HB-HN7–18) > 41.52 KDa (HB-HN1–3) > 39.67 KDa (YN-MRS-17), however, electrophoretic mobility speed of each protein on SDS-PAGE seems not related with the protein size, which is HB-HN6–8 < HB-HN7–18 < HB-HN9–3 < LN-AP-1 < HB-HN1–3 < YN-MRS-17.

Three polyclonal antibodies PAb-HB-HN9–3, PAb-HB-HN6–8 and PAb-YN-MRS-17 were raised against purified rCPs (HB-HN9–3, HB-HN6–8 and YN-MRS-17, respectively). The six rCPs were analyzed by western blot (Fig. 2b, c and d) with the three different antibodies. Hybridization signals on the blots were quantified using Image J software to compare the serological reactivity between rCPs. Results showed that the six rCPs could react with each of the three antibodies, but the reaction signals intensities between each antibody and rCP combinations were different, in general, we found that the reaction intensity was positively correlated to CP amino acids similarity between different isolates. For example, HB-HN6–8 (E/Gp1), YN-MRS-17 (D/Gp1) and HB-HN9–3 (F/Gp2) shared the lowest amino acid sequence identity with themselves, and we found the reaction intensities between each antibody and their own original rCP are strongest. HB-HN6–8, YN-MRS-17 and HB-HN9–3 shared the lowest amino acid sequence identity with HB-HN9–3, HB-HN9–3 and HB-HN6–8, respectively, and we found these antibodies and rCP combinations has the weakest signals. Our results indicated that genetic variation of CP variants results in differences in serological reactivity, probably due to differences in epitopes.

### ASPV CPs different in their propensity to aggregate

**Fig. 3:**
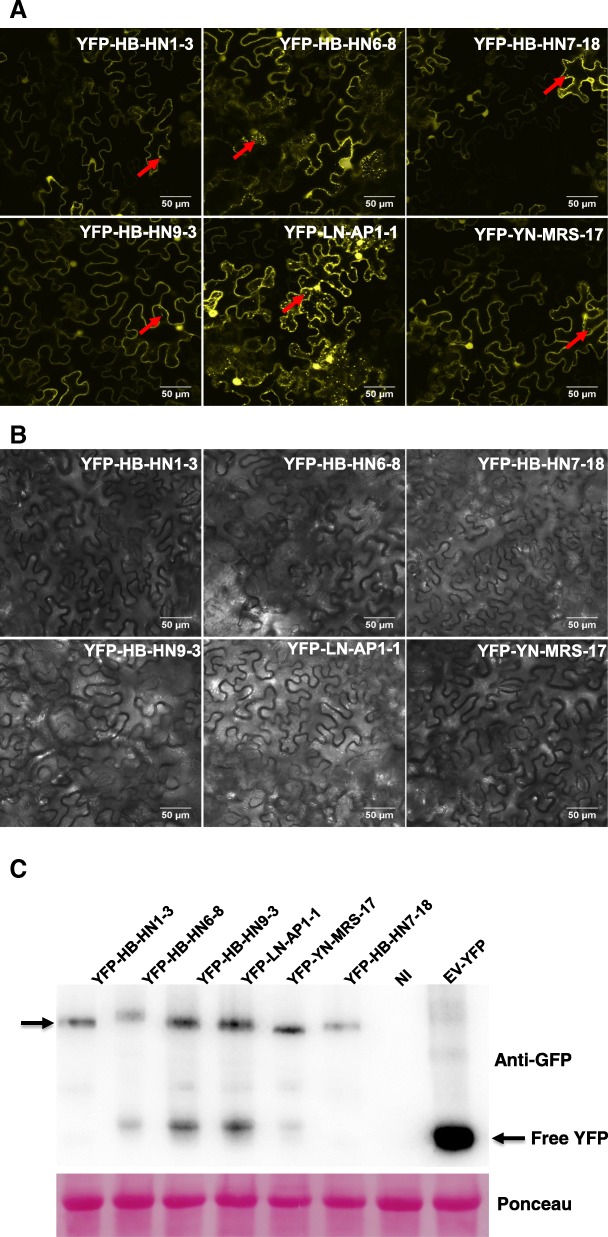
ASPV CPs differ in their propensity to aggregate. Subcellular localization of YFP-ASPV-CPs upon transient expression in *Nicotiana. benthamiana* leaves. Confocal micrographs of *N. benthamiana* leaf cells expressing YFP-ASPV-CPs (as indicated at the top left of each image) were taken at 72 hpi (Scale Bar, 50 μm). **a** YFP channel. **b** TD channel. Red arrowheads indicate inclusions. The results shown are representative of three separate experiments. **c** Total protein was extracted from YFP-ASPV-CPs infiltrated leaf spots. Samples were subjected to anti-GFP immune-blotting. Ponceau staining are shown as a loading control

To elucidate whether genetic diversity of ASPV CPs affects their subcellular localization in plant cell, we next examined subcellular localization of CP variants by transiently expressing CPs fused with YFP at its N-terminus (YFP-ASPV-CPs (HB-HN1–3/HB-HN9–3/HB-HN6–8/HB-HN7–18/YN-MRS-17/LN-AP1–1)) in *N. benthamiana*, which is widely used to express protein transiently [25]. As shown in Fig. 3a and b, when expressed alone, the six YFP-ASPV CPs localized to the plasmodesmata (PD) and formed intensely fluorescent foci (aggregates/inclusions) in the cytoplasm. However, the degree to which the ASPV CPs formed aggregates differed, with YFP-LN-AP1–1, isolated from apple, forming the most aggregates, followed by YFP-HB-HN6–8, a pear isolate belonging to subgroup E [13], whereas the protein expression level of these CPs variants does not have a great difference (Fig. 3c). This difference in the aggregating propensity of the six CPs may be the result of local structural changes introduced by the different amino acids changes in the N terminus.

### ASPV CP possesses VSR activity

**Fig. 4:**
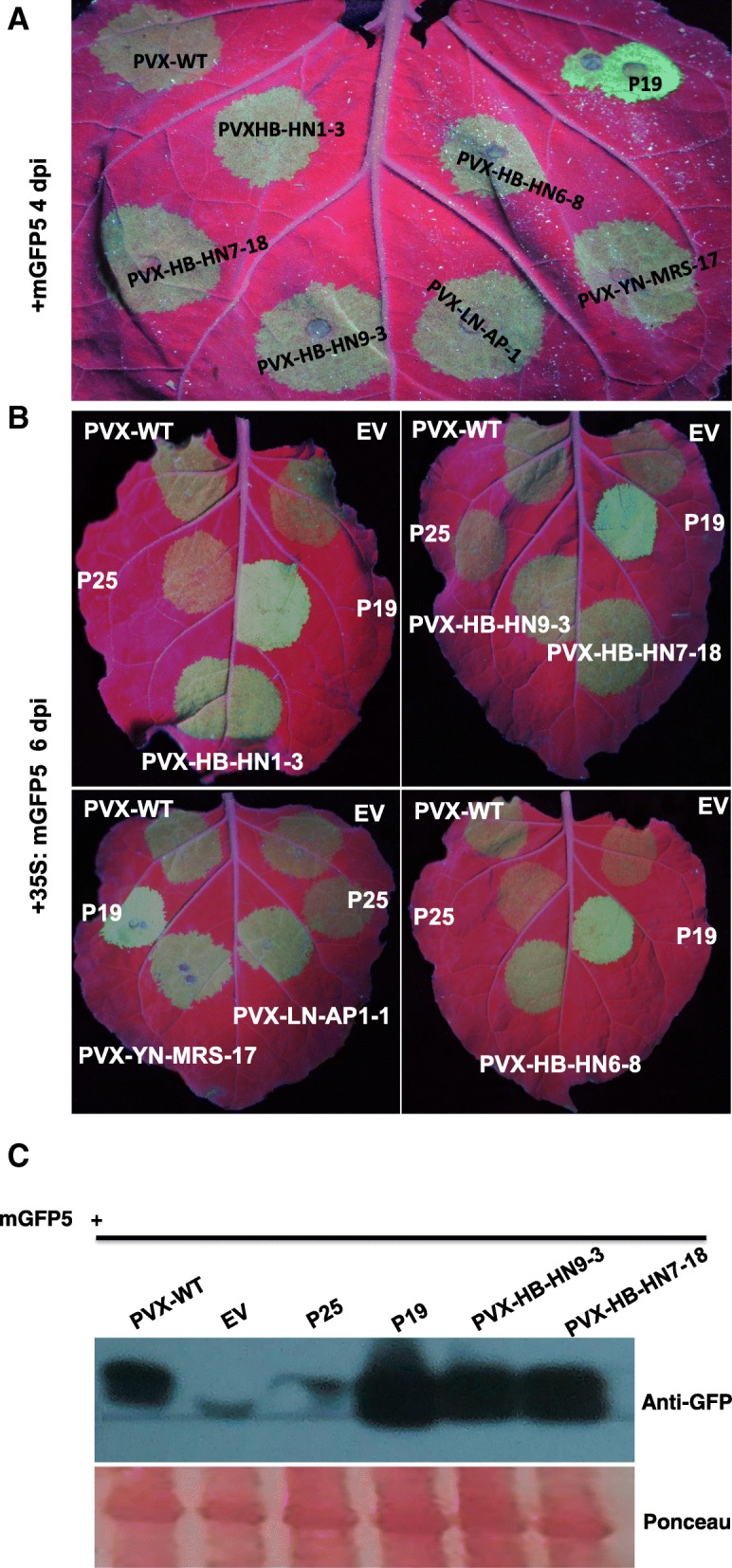
ASPV CPs possess VSR activity. Local patches on *N. benthamiana* leaf were agroinfiltrated with 35S:mGFP5 in combination with either 35S:P19, 35S:P25, PVX (wt) or PVX-ASPV-CPs, respectively. **a** PVX-ASPV-CPs were infiltrated on the same leaf. **b** PVX-ASPV-CPs were infiltrated on different leaves. GFP signal was monitored by UV illumination at 4 days post infiltration (dpi) (**a**) and 6 dpi (**b**). **c** Total protein was extracted from leaf infiltration spots of (**b**). Samples were subjected to anti-GFP immune-blotting. Ponceau staining are shown as a loading control. All experiments were repeated 3 times and representative results are shown

**Fig. 5:**
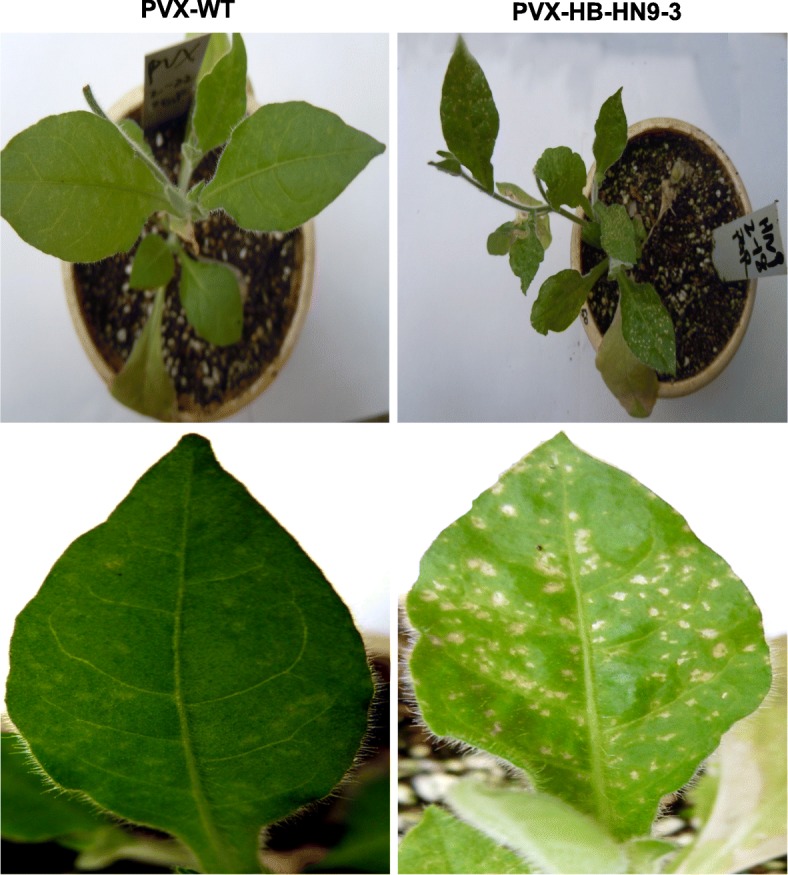
ASPV CPs induce symptoms on *N. occidentalis.* Five-week-old *N. occidentalis* plants were agroinfiltrated with PVX (wt) or PVX-ASPV-CPs and symptoms induced on these plants were photographed at 30 dpi

The ASPV TGBp1 is homologous to the PVX P25 protein, which possesses VSR activity that suppresses systemic RNA silencing but not local RNA silencing [26]. Here, transient expression of 35S:mGFP5 in combination with 35S: ASPV-TGB1, TGB2 or TGB3 in wild type *N. benthamiana* or the GFP-expressing transgenic line 16c leaves (which are used to test local and systemic VSR activity, respectively) [26] results in bright fluorescence visible at 3 dpi. However, at 4 dpi GFP expression was silenced and fluorescence was only faintly visible in infiltrated leaves (Additional file 2: Figure S1 A). At the same time systemic GFP fluorescence in 16c plants became silenced at 14 dpi (Additional file 2: Figure S1 B). These results indicate that none of the ASPV TGB proteins possess VSR activity. However, co-expression of 35S:mGFP5 with PVX-HB-HN1–3/HB-HN9–3/HB-HN6–8/HB-HN7–18/YN-MRS-17/LN-AP1–1 resulted in stronger GFP fluorescence at 6 dpi in *N. benthamiana* compared to 35S:mGFP5 with PVX (wt) (Fig. 4a and b). The increased GFP accumulation was further confirmed by western-blot (Fig. 4c). These results suggested that the ASPV CP possesses VSR activity, and that the VSR activity of different CP variants displayed no obvious differences. Previous studies have indicated that virus-encoded VSRs often act as pathogenic determinants [27]. To test whether ASPV CP affected pathogenicity, *N. occidentalis* plants were infected with PVX-CPs and with PVX (wt). Infection of *N. occidentalis* plants with PVX-CPs resulted in more serious symptoms at 30 dpi compared to PVX (wt) infected plants (Fig. 5). This result indicated that ASPV CP is a pathogenic determinant, although we did not observe different pathogenic ability between different CPs.

## Discussion

In this study, we have shown that ASPV isolates originating from pears with or without symptoms (Fig. 1a) or symptomless apple plants in the same orchard (Wuhan, China) induced different symptoms in *N. occidentalis* (Fig. 1b). Viral infections can induce symptoms by interacting and interfering with host components [28], and viral CPs have often been implicated in contributing to infectivity, pathogenicity, and symptom development [14, 29]. Our result showing that each PVX-ASPV-CP enhanced PVX symptoms in *N. occidentalis* implied that ASPV CP also functions as a symptom determinant (Fig. 5). This may be related to its VSR activity as similar increases in symptoms have been observed when VSRs from other viruses are expressed from the PVX genome [30–34]. We do not rule out other possibility, but it seems that distinct characteristics of ASPV CP variants may account for the different symptoms induced by ASPV isolates on *N. occidentalis*.

First, we have previously shown that CP variant composition of these isolates is complicated and sequences of CP variants from subgroups have great differences [13]. For example, isolate HB-HN9 consists of variants from subgroup E and F, HB-HN7 (subgroup A and B), HB-HN1/HB-HN2 (subgroup A), HB-HN6/HB-HN10 (subgroup E), HB-AP1 (Apple group) [13]. Pervious study showed that viral CP modifications lead to symptom changes, fifteen Pepino mosaic potexvirus (PepMV) isolates that shared a very close similarity in their CP sequences caused similar symptoms, while one strain that has great differences in the CP sequence did not induce symptoms in tomato [35]. The N-terminus of ASPV CP variants belonging to different subgroups have been shown to have high variability in length and sequence [13], thus it is likely that N-terminal of ASPV CP variants is differently involved in interactions with host factors. This is not only the case of ASPV CP, as several reports have shown that differences in N-terminus of viral CPs results in differences in CP functions: The N-terminus of Potato Virus Y (PVY) CP plays a critical role in symptom development and determines the pathogenicity of different PVY isolates [36]. In *Arabidopsis* Col-0 and WS plants, the systemic infection of Tobacco Etch potyvirus (TEV) was restricted by RTM genes (Restricted TEV Movement, RTM1, RTM2, and RTM3) through interaction with TEV CP, with amino acid changes in the CP N-terminus resulting in resistance-breaking strains of TEV [37, 38]. The N-terminus of the Plum pox potyvirus (PPV) CP is likely important to virus systemic movement, probably via effects on virion assembly and/or stability; modifications in the N-terminal 30 aa region of two PPV CP variants results in systemic infection of *N. benthamiana* and *N. clevelandii* by PPV [39]. The molecular basis of this link is not yet clear, although it is possible that differences in CP sequences affect viral transport or virion assembly and/or stability in plant cell.

Consistent with the above examples, we next showed that in the absence of ASPV, YFP-CPs variants have different abilities to form aggregates in the cytoplasm of infiltrated *N. benthamiana* cells at 72 hpi, with variant LN-AP1–1 forming the most aggregates, followed by HB-HN6–8. Viral proteins form aggregates in the cytoplasm and nuclei of infected cells [40]. However, the role of viral proteins aggregates remains dynamic: viruses may use aggregates to concentrate host and viral proteins in one place in the cell to facilitate replication, assembly and movement. Alternatively, aggregates may form part of an innate cellular immunity response that recognizes virus components and targets them for storage and degradation [40]. A previous study showed that Tomato yellow leaf curl virus (TYLCV) CP forms aggregates of increasing size during viral infection, suggested a functional role for aggregates during the development of infection [41]. If the same were true for ASPV CP aggregates, we would infer that the CP variant (LN-AP1–1), which has a stronger aggregate formation propensity, could recruit more viral and host components required for viral replication and assembly in the same infection time. As a result, viral isolates that harbor this kind of CP variant would replicate efficiently during early infection stages and more virus progeny would appear in infected plant cells. Unfortunately, this could not be further explored due to a lack of ASPV infectious clone, which needs to be done in future work. Since the CP of each ASPV isolate contained two or more variants in pear or apple plants, and different variants have different aggregation propensity, which then probably results in different infection efficiency. This result implies that the different symptoms induced by ASPV isolates in *N. occidentalis* may be affected by different CPs variants due to the intrinsic propensity to aggregate. Our study of different ASPV CP variants aggregation propensity in plant cells provides new insights into virus-host interactions. RNA silencing is one of the major documented antiviral mechanism that inhibits RNA viruses and RNA viruses encode VSRs as a strategy to defend against RNA silencing. However, in contrast to *Potexvirus* encoded TGBp1, our results indicate that TGBp1 encoded by ASPV, which is a member of *Foveavirus*, is neither a local nor a systemic silencing suppressor (Additional file 2: Figure S1). Interestingly, ASPV CP, when expressed in PVX vector, suppressed local silencing in *N. benthamiana* (Fig. 4) and induced more serious symptoms in *N. occidentalis* compared to PVX (wt) (Fig. 5). Several lines of evidence support our result, firstly, proteins homologous to TGB1 encoded by members of different viral genera do not necessarily have the same functions. For example, TGBp1 encoded by Barley stripe mosaic virus (BSMV, genus *Hordeivirus*) or Peanut clump virus (PCV, genus *Pecluvirus*) does not function as a VSR, instead, γb of BSWV and p15 encoded by PCV supressed RNA silencing [42, 43]; Potato virus M (PVM), a member of the genus *Carlavirus*, employs a dual strategy to block host antiviral silencing, TGBp1 showed suppressor activity only on systemic silencing, whereas a cysteine-rich protein (CRP) encoded by PVM inhibited both local and systemic silencing [44]; even in the genus *Potexviruse*, the ability of TGBp1 encoded by *potexviruses* to suppress RNA silencing varies significantly in *N. benthamiana*: PVX TGBp1 (p25) is one of the well-characterized VSR, supressed systemic silencing but not local silencing [26]; TGBp1 encoded Tulip virus X (TVX) showed very weak (almost no) local silencing ability [16], wherein TGBp1 of *Plantago asiatica* mosaic virus (PlAMV), Asparagus virus 3 (AV3), and White clover mosaic virus (WClMV) have very strong local and systemic silencing suppressor activity [16]. Secondly, the CP encoded by several plant viruses have been shown to be a VSR, for example, Cowpea mosaic virus (CPMV), Tomato ringspot virus (ToRSV), Cocksfoot mottle virus (CfMV) [45–47]. Likewise, in the case of the Potexvirus PepMV, in addition to the TGB1 protein, the CP also showed strong VSR activity [48]. Third, many VSRs have been identified as pathogenicity determinants or symptom determinants [49] and consistent with this, we showed that the ASPV CP is also a symptom determinant (Fig. 5).

In addition, we showed that RNA silencing suppressing ability of six ASPV CP variants that differed greatly at their N termini, turned out to be the same (Fig. 4). This is not a surprise, since all identified VSRs are diverse in sequence and structure, and act via different molecular mechanisms [50]. How ASPV CPs variants maintain their conserved VSR activity remains unknown in this study, although it is possible that the conserved C-terminal of ASPV CP mediates its VSR ability [13]. The sequencing of the pear (*Pyrus bretschneideri* Rehd.) genome [51] and the identification of some RNA silencing components in pear provide a great opportunity to further study the antiviral role of pear RNA silencing components [52] and how ASPV CP functions in counteracting the RNA silencing pathway.

Different levels of serological reactivity among the tested six CPs with the three antibodies (Fig. 2) underlined the necessity of producing different antibodies according to different ASPV isolates. ASPV diagnosis in the field, at least in China, would benefit from our work. Also, considering the 3′ terminal region of the ASPV CP is relatively conserved, in the future, it would be more useful to raise antibodies against the more conserved part of ASPV CP.

## Conclusions

Taken together, our results indicate that the ASPV CP has VSR activity, which is conserved among the different CP variants. However, CP variants have different abilities to aggregate in *N. benthamiana*, probably due to amino acid differences in the N terminus, which may also result in different symptoms induced by ASPV isolates in *Nicotiana. occidental*. Until now, little has been known about the molecular defense and counter defense mechanisms between ASPV and its host, however, our study provides insights that can be used to further explore this topic.

## Additional files

Additional file 1: **Table S1** List of primers used in this study. (DOCX 18 kb)

Additional file 2: **Figure S1** ASPV TGB proteins do not affect local or systemic RNA silencing. A, *Nicotiana benthamiana* leaf patches were agroinfiltrated with 35S:mGFP5 in combination with 35S:EV, 35S:P19, 35S:P25, 35S:ASPV-TGB1, 35S:ASPV-TGB2 or 35S:ASPV-TGB3, as indicated. GFP fluorescence was monitored by UV illumination at 4 dpi. B, Two leaves per 16c N. benthamiana plant were agroinfiltrated with 35S:mGFP5 in combination with 35S:EV, 35S:P19, 35S:P25, 35S:ASPV-TGB1, 35S:ASPV-TGB2 or 35S:ASPV-TGB3, as indicated. GFP fluorescence was monitored in systemic leaves by UV illumination at 14 dpi. **Figure S2** Antibodies PAb-HB-HN6–8, PAb-YN-MRS-17 and PAb-HB-HN9–3 were used to detect ASPV-CPs expressed from a PVX vector in *Nicotiana occidentalis* plants. A-C, Total protein was extracted from PVX-ASPV-CPs infected *N. occidentalis* plants, as indicated. Samples were subjected to antibody PAb-HB-HN6–8, PAb-YN-MRS-17 and PAb-HB-HN9–3 immune-blotting, respectively. (PPTX 861 kb)

## Abbreviations

AP: Alkaline phosphatase
ASPV: Apple stem pitting virus
AV3: Asparagus virus 3
BSMV: Barley stripe mosaic virus
CfMV: Cocksfoot mottle virus
CP: Coat protein
CPMV: Cowpea mosaic virus
CTAB: Cetyltriethylammonium bromide
dpi: Days post inoculation/infiltration
IPTG: Isopropyl-β-d-thiogalactoside
LB: Luria-Bertani
PCV: Peanut clump virus
PD: Plasmodesmata
PepMV: Pepino mosaic potexvirus
PlAMV: *Plantago asiatica* mosaic virus
PPV: Plum pox potyvirus
PVM: Potato virus M
PVX: Potato Virus X
PVY: Potato Virus Y
RTM: Restricted TEV Movement
RT-PCR: Reverse Transcription -Polymerase Chain Reaction
TEV: Tobacco Etch potyvirus
TGB: Triple gene block proteins
ToRSV: Tomato ringspot virus
TVX: Tulip virus X
TYLCV: Tomato yellow leaf curl virus
UTR: Untranslated region
VSR: Suppressor of RNA silencing
WClMV: White clover mosaic virus
