# Supplementary material for: Functional analysis of apple stem pitting virus coat protein variants
Source: Virol J. 2019 Feb 8;16:20. doi: 10.1186/s12985-019-1126-8 (PMC6368714; doi:10.1186/s12985-019-1126-8)
Supplement: Supplementary file 1 — Table S1 List of primers used in this study. (DOCX 18 kb) [file 12985_2019_1126_MOESM1_ESM.docx]

Additional fie 1: **Table S1.** List of primers used in this study.

| **Construct name** | **Primers with Restriction Enzyme cutting site (5'-3')** | **Restriction**  **Enzyme** |  |
| --- | --- | --- | --- |
| pET-HB-HN9-3 | *Forward*: GG*GGATCC*ATGGCTTCCGATGGTACT | *Bam*HI |  |
|  | *Reverse*: GGG*AAGCTT*TCACTTCCTAATTGATAG | *Hin*dI |  |
| pET-HB-HN1-3 | *Forward*: GGG*GAGCTC*ATGGCATCCGATGGCTCT | *Sac*I |  |
|  | *Reverse*: GG*GTCGAC*TTACTTCTTAATGGATAG | *Sal*I |  |
| pET-HB-HN6-8 | *Forward*: GGG*GAGCTC*ATGGCTTCCAATGTATCC | *Sac*I |  |
|  | *Reverse*: GG*GTCGAC*TTACTTCCTAATGGATAA | *Sal*I |  |
| pET-HB-HN7-18 | *Forward*: GGG*GAGCTC*ATGACTTCCAATGGTTCC | *Sac*I |  |
|  | *Reverse*: GG*GTCGAC*TTACTTCCTAATGGATAA | *Sal*I |  |
| pET-YN-MRS-17 | *Forward*: GGG*GAGCTC*ATGACTTCTAATGGATCC | *Sac*I |  |
|  | *Reverse*: GG*GTCGAC*TTACTTCCTAATGGATAG | *Sal*I |  |
| pET-LN-AP1-1 | *Forward*: GGG*GAGCTC*ATGGCTTCCAATGGTTCC | *Sac*I |  |
|  | *Reverse*: GG*GTCGAC*TTACTTCCTGATGGATAG | *Sal*I |  |
| PVX-HB-HN9-3 | *Forward*: GGATCGATATGGCTTCCGATGGTACT | *Cla*I |  |
|  | *Reverse*: GGGTCGACTCACTTCCTAATTGATAG | *Sal*I |  |
| PVX-HB-HN1-3 | *Forward*: GGATCGATATGGCATCCGATGGCTCT | *Cla*I |  |
|  | *Reverse*: GGGTCGACTTACTTCTTAATGGATAG | *Sal*I |  |
| PVX-HB-HN6-8 | *Forward*: GGATCGATATGGCTTCCAATGTATCC | *Cla*I |  |
|  | *Reverse*: GGGTCGACTTACTTCCTAATGGATAA | *Sal*I |  |
| PVX-HB-HN7-18 | *Forward*: GGATCGATATGACTTCCAATGGTTCC | *Cla*I |  |
|  | *Reverse*: GG*GTCGAC*TTACTTCCTAATGGATAA | *Sal*I |  |
| PVX-YN-MRS-17 | *Forward*: GGATCGATATGACTTCTAATGGATCC | *Cla*I |  |
|  | *Reverse*: GG*GTCGAC*TTACTTCCTAATGGATAG | *Sal*I |  |
| PVX-LN-AP1-1 | *Forward*: GGATCGATATGGCTTCCAATGGTTCC | *Cla*I |  |
|  | *Reverse*: GG*GTCGAC*TTACTTCCTGATGGATAG | *Sal*I |  |
| pEAQ-YFP-HB-HN9-3 | *Forward*: GG*GCTAGC*ATGGCTTCCGATGGTACT | *Nhe*I |  |
|  | *Reverse*: GG*GTCGAC* TCACTTCCTAATTGATAG | *Sal*I |  |
| pEAQ-YFP-HB-HN1-3 | *Forward*: GCTCTAGAATGGCATCCGATGGCTCT | *Xba*I |  |
|  | *Reverse*: GG*GTCGAC*TTACTTCTTAATGGATAG | *Sal*I |  |
| pEAQ-YFP-HB-HN6-8 | *Forward*: GCTCTAGAATGGCTTCCAATGTATCC | *Xba*I |  |
|  | *Reverse*: GG*GTCGAC*TTACTTCCTAATGGATAA | *Sal*I |  |
| pEAQ-YFP-HB-HN7-18 | *Forward*: GCTCTAGAATGACTTCCAATGGTTCC | *Xba*I |  |
|  | *Reverse*: GG*GTCGAC*TTACTTCCTAATGGATAA | *Sal*I |  |
| pEAQ-YFP-YN-MRS-17 | *Forward*: GGG*GCTAGC*ATGACTTCTAATGGATCC | *Nhe*I |  |
|  | *Reverse*: GG*GTCGAC*TTACTTCCTAATGGATAG | *Sal*I |  |
| pEAQ-YFP-LN-AP1-1 | *Forward*: GCTCTAGA ATGGCTTCCAATGGTTCC | *Xba*I |  |
|  | *Reverse*: GG*GTCGAC* TTACTTCCTGATGGATAG | *Sal*I |  |
| pBIN61-ASPV-TGB1 | | *Forward*: GCTCTAGAATGGAAACTGTGCTCAGTTTG | *Xba*I |
|  |  | *Reverse*: CGCGGATCCATTAGTCACAAGCACGAGC | *BamH*I |
| pBIN61-ASPV-TGB2 | | *Forward*: GCTCTAGAATGCCTCTTGCTCAACCC | *Xba*I |
|  |  | *Reverse*: CGCGGATCCCTCGTGTACAGTTGCTG | *BamH*I |
| pBIN61-ASPV-TGB3 | | *Forward*: GCTCTAGAATGTTTCCGAGAAGTGGAGTG | *Xba*I |
|  |  | *Reverse*: CGCGGATCCACCTAATGGGTGGTAATA | *BamH*I |
