# Supplementary material for: Functional analysis of apple stem pitting virus coat protein variants
Source: Virol J. 2019 Feb 8;16:20. doi: 10.1186/s12985-019-1126-8 (PMC6368714; doi:10.1186/s12985-019-1126-8)
Supplement: Supplementary file 2 — Figure S1 ASPV TGB proteins do not affect local or systemic RNA silencing. A, Nicotiana benthamiana leaf patches were agroinfiltrated with 35S:mGFP5 in combination with 35S:EV, 35S:P19, 35S:P25, 35S:ASPV-TGB1, 35S:ASPV-TGB2 or 35S:ASPV-TGB3, as indicated. GFP fluorescence was monitored by UV illumination at 4 dpi. B, Two leaves per 16c N. benthamiana plant were agroinfiltrated with 35S:mGFP5 in combination with 35S:EV, 35S:P19, 35S:P25, 35S:ASPV-TGB1, 35S:ASPV-TGB2 or 35S:ASPV-TGB3, as indicated. GFP fluorescence was monitored in systemic leaves by UV illumination at 14 dpi. Figure S2 Antibodies PAb-HB-HN6–8, PAb-YN-MRS-17 and PAb-HB-HN9–3 were used to detect ASPV-CPs expressed from a PVX vector in Nicotiana occidentalis plants. A-C, Total protein was extracted from PVX-ASPV-CPs infected N. occidentalis plants, as indicated. Samples were subjected to antibody PAb-HB-HN6–8, PAb-YN-MRS-17 and PAb-HB-HN9–3 immune-blotting, respectively. (PPTX 861 kb) [file 12985_2019_1126_MOESM2_ESM.pptx]

## Slide 1
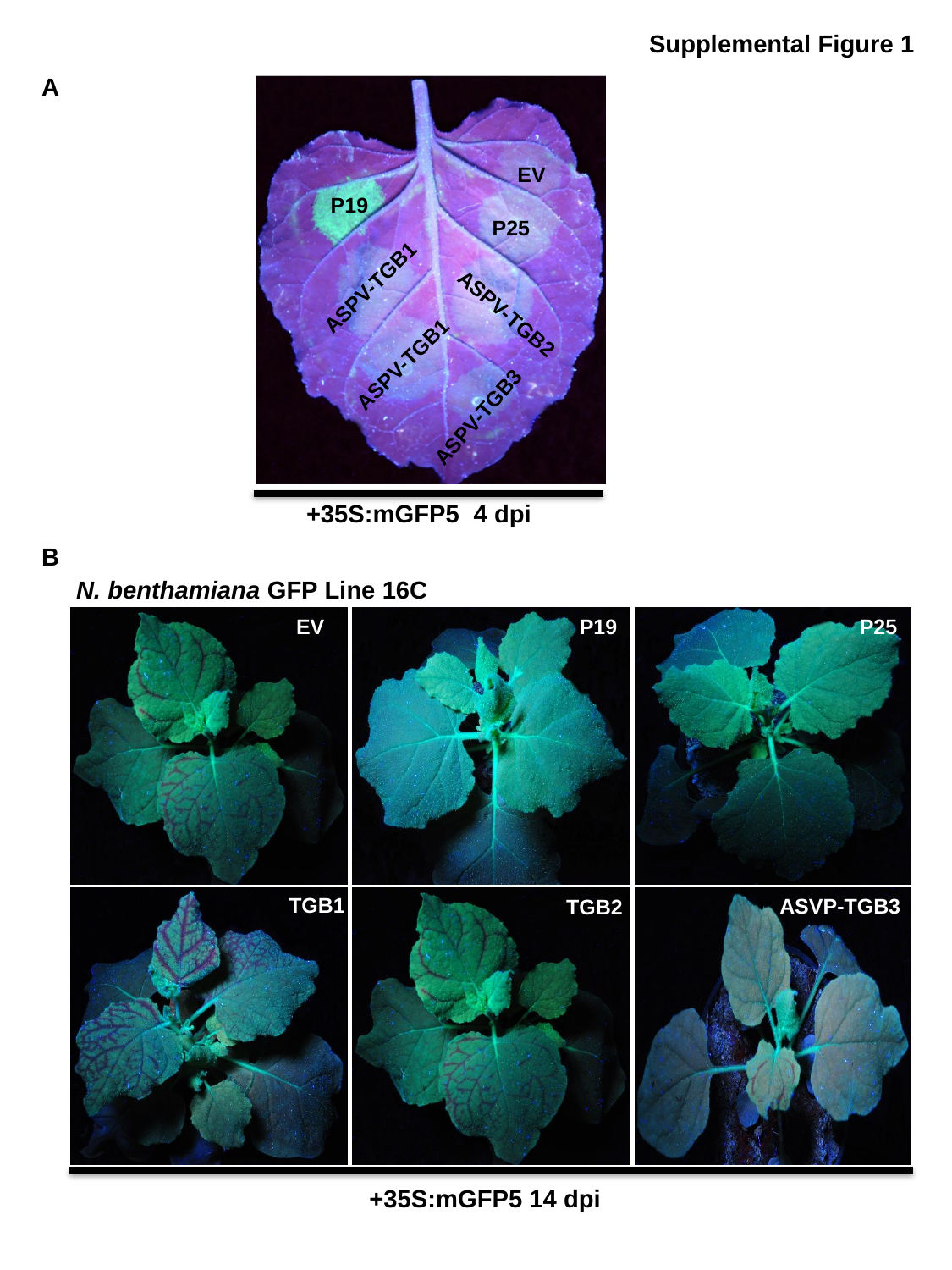

Supplemental Figure 1
A
EV
P19
P25
ASPV-TGB1
ASPV-TGB2
ASPV-TGB1
ASPV-TGB3
+35S:mGFP5 4 dpi
B
N. benthamiana GFP Line 16C
P19
P25
EV
TGB1
ASVP-TGB3
TGB2
+35S:mGFP5 14 dpi

## Slide 2
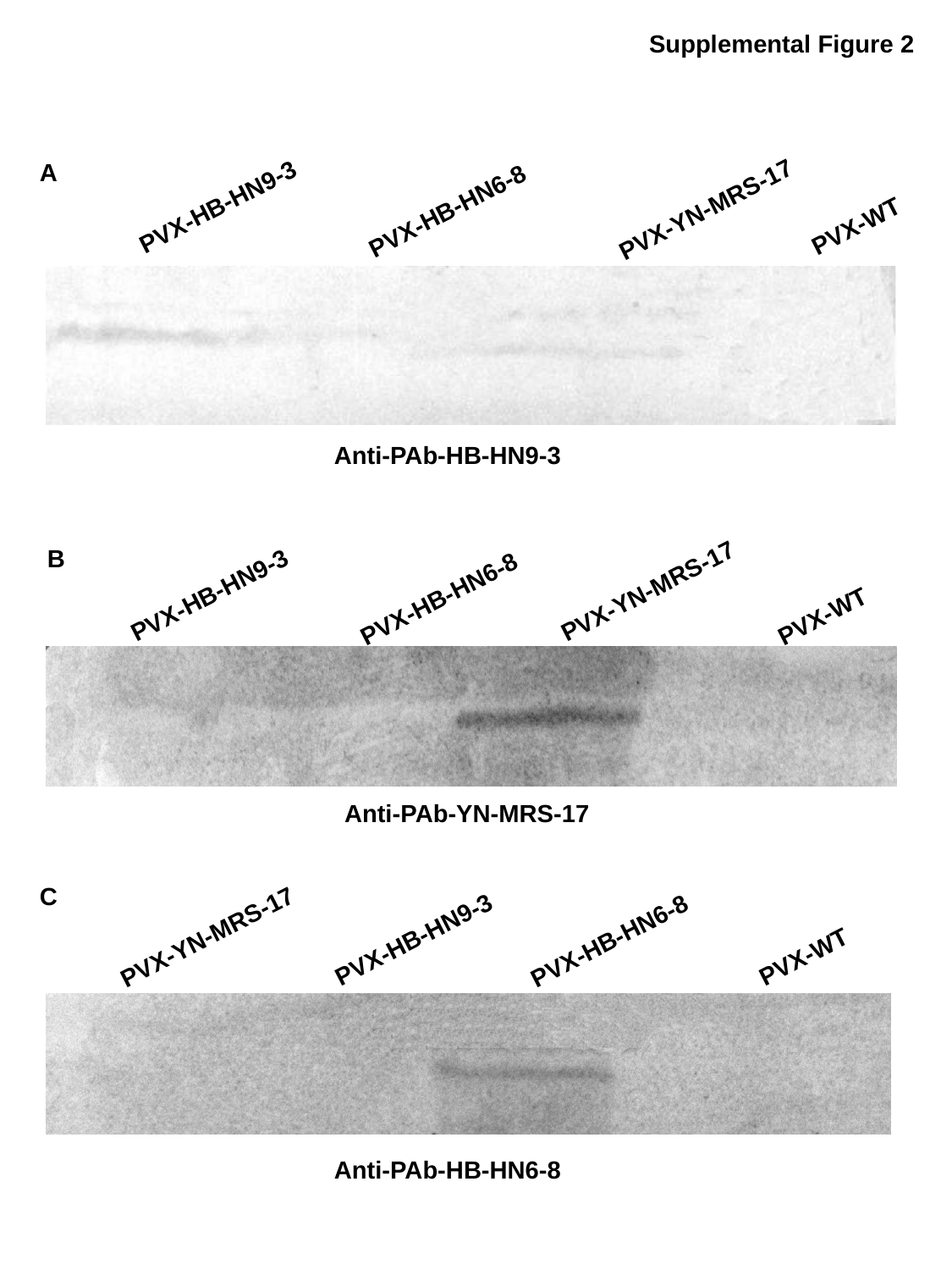

Supplemental Figure 2
A
 PVX-HB-HN6-8
 PVX-HB-HN9-3
 PVX-YN-MRS-17
 PVX-WT
Anti-PAb-HB-HN9-3
B
 PVX-YN-MRS-17
 PVX-HB-HN6-8
 PVX-HB-HN9-3
 PVX-WT
Anti-PAb-YN-MRS-17
C
 PVX-YN-MRS-17
 PVX-HB-HN6-8
 PVX-HB-HN9-3
 PVX-WT
Anti-PAb-HB-HN6-8
